# Release of Lungworm Larvae from Snails in the Environment: Potential for Alternative Transmission Pathways

**DOI:** 10.1371/journal.pntd.0003722

**Published:** 2015-04-17

**Authors:** Alessio Giannelli, Vito Colella, Francesca Abramo, Rafael Antonio do Nascimento Ramos, Luigi Falsone, Emanuele Brianti, Antonio Varcasia, Filipe Dantas-Torres, Martin Knaus, Mark T. Fox, Domenico Otranto

**Affiliations:** 1 Department of Veterinary Medicine, University of Bari, Valenzano, Bari, Italy; 2 Department of Veterinary Science, University of Pisa, Pisa, Italy; 3 Department of Veterinary Science, University of Messina, Messina, Italy; 4 Department of Veterinary Medicine, University of Sassari, Sassari, Italy; 5 Department of Immunology, Aggeu Magalhães Research Institute, Oswaldo Cruz Foundation, Recife, Pernambuco, Brazil; 6 Merial GmbH, Kathrinenhof Research Center, Rohrdorf, Germany; 7 Department of Pathology and Pathogen Biology, The Royal Veterinary College, University of London, London, United Kingdom; George Washington University School of Medicine and Health Sciences, UNITED STATES

## Abstract

**Background:**

Gastropod-borne parasites may cause debilitating clinical conditions in animals and humans following the consumption of infected intermediate or paratenic hosts. However, the ingestion of fresh vegetables contaminated by snail mucus and/or water has also been proposed as a source of the infection for some zoonotic metastrongyloids (e.g., *Angiostrongylus cantonensis*). In the meantime, the feline lungworms *Aelurostrongylus abstrusus* and *Troglostrongylus brevior* are increasingly spreading among cat populations, along with their gastropod intermediate hosts. The aim of this study was to assess the potential of alternative transmission pathways for *A*. *abstrusus* and *T*. *brevior* L3 *via* the mucus of infected *Helix aspersa* snails and the water where gastropods died. In addition, the histological examination of snail specimens provided information on the larval localization and inflammatory reactions in the intermediate host.

**Methodology/Principal Findings:**

Twenty-four specimens of *H*. *aspersa* received ~500 L1 of *A*. *abstrusus* and *T*. *brevior*, and were assigned to six study groups. Snails were subjected to different mechanical and chemical stimuli throughout 20 days in order to elicit the production of mucus. At the end of the study, gastropods were submerged in tap water and the sediment was observed for lungworm larvae for three consecutive days. Finally, snails were artificially digested and recovered larvae were counted and morphologically and molecularly identified. The anatomical localization of *A*. *abstrusus* and *T*. *brevior* larvae within snail tissues was investigated by histology. L3 were detected in the snail mucus (i.e., 37 *A*. *abstrusus* and 19 *T*. *brevior*) and in the sediment of submerged specimens (172 *A*. *abstrusus* and 39 *T*. *brevior*). Following the artificial digestion of *H*. *aspersa* snails, a mean number of 127.8 *A*. *abstrusus* and 60.3 *T*. *brevior* larvae were recovered. The number of snail sections positive for *A*. *abstrusus* was higher than those for *T*. *brevior*.

**Conclusions:**

Results of this study indicate that *A*. *abstrusus* and *T*. *brevior* infective L3 are shed in the mucus of *H*. *aspersa* or in water where infected gastropods had died submerged. Both elimination pathways may represent alternative route(s) of environmental contamination and source of the infection for these nematodes under field conditions and may significantly affect the epidemiology of feline lungworms. Considering that snails may act as intermediate hosts for other metastrongyloid species, the environmental contamination by mucus-released larvae is discussed in a broader context.

## Author Summary

Gastropod-borne parasites may cause debilitating clinical conditions in animals and humans. The infection occurs by consumption of intermediate hosts (i.e., snails). However, the ingestion of fresh vegetables contaminated by water and/or snail mucus has been proposed as a transmission source for some zoonotic metastrongyloids (e.g., *Angiostrongylus cantonensis*). *Aelurostrongylus abstrusus* and *Troglostrongylus brevior* cause severe clinical conditions in cats and their prevalence is increasing in feline populations. The epidemiology of feline lungworm infections is poorly understood and the role of gastropod molluscs, as vectors of the disease, is questioned. Since metastrongyloid nematodes may use other little-studied infection pathways, the presence of *A*. *abstrusus* and *T*. *brevior* larvae was herein assessed in the mucus of *Helix aspersa* snails and in the water where specimens died. The finding of *A*. *abstrusus* and *T*. *brevior* infective L3 in the mucus shed by *H*. *aspersa* or when infected gastropods die submerged in water indicates that alternative pathways for the transmission of feline lungworms may occur. Accordingly, these results should spur the interest of the scientific community towards the delineation of further infection routes for other metastrongyloids of major human concern, such as *Angiostrongylus costaricensis* and *A*. *cantonenis*.

## Introduction

Gastropod-borne agents infect approximately 300 million people worldwide, causing major debilitating conditions, as well as adversely affecting quality of life and healthcare. For example, some ailments may contribute to the development of neoplastic tumours, such as cholangiocarcinoma during opisthorchiosis/clonorchiosis [1] or may induce debilitating diseases (e.g., schistosomiosis or fascioliosis) [2,3]. In addition, human beings may act as dead-end hosts for zoonotic metastrongyloids of rats, such as *Angiostrongylus cantonensis* or *Angiostrongylus costaricensis* (Strongylida, Angiostrongylidae), which cause eosinophilic meningitis and ileitis, respectively [4,5]. The main pathway for human infection is represented by the consumption of undercooked intermediate hosts (i.e., snails) or raw paratenic hosts (e.g., prawns and freshwater shrimps) [6,7].

In veterinary medicine, the lungworm *Aelurostrongylus abstrusus* (Strongylida, Angiostrongylidae), along with the less-known *Troglostrongylus brevior* (Strongylida, Crenosomatidae) are of increasing concern due to their spreading in cat populations [8–10]. Indeed, these parasites share a similar biology and ecological niches, and may infect the same individual [10,11]. Although gastropod molluscs are recognized as the intermediate hosts for feline lungworms [12], the role of snails in the transmission of the infection is debatable because they are not preferred preys for felids [13,14]. As a result, paratenic hosts (i.e., rodents, birds, amphibians and reptiles) may play a crucial role in the epidemiology of *A*. *abstrusus* [15,16]. Metastrongyloid nematodes may, however, use additional routes of the infection, as it has been hypothesized for the vertical transmission of *T*. *brevior* in cats [17].

Land snails excrete a thin layer of pedal mucus, consisting of water and mucin-like carbohydrate-protein complexes, which acts as glue and lubricant during the locomotion [18]. Again, the production of the mucous trail facilitates several functions of the snail, such as the homing behaviour, aggregation, and protection against desiccation and predation [18]. Interestingly, gastropods may be able to release mucous secretions of variable quality, depending on the nature of the external stimuli (i.e., mechanical or chemical) [19]. The ingestion of fresh vegetables, contaminated by water and/or snail mucus harbouring *A*. *cantonensis* third-stage larvae (L3), has been previously suggested in a recent outbreak of human eosinophilic meningitis in Jamaica [20]. Accordingly, the presence of *A*. *cantonensis* and *A*. *costaricensis* in the mucus of slugs or snails and in contaminated water has been investigated, but their role in the epidemiology of the infection is yet to be confirmed [7].

This study sought to establish whether *A*. *abstrusus* and *T*. *brevior* L3 could be found in the mucus of infected *Helix aspersa* snails and in the water where specimens had died. Larval localization in snails and inflammatory reactions in the intermediate hosts has also been investigated by histology.

## Materials and Methods

### Snail maintenance and infection

Adult *H*. *aspersa* (n = 200) were sourced from a commercial farming centre in Barletta (southern Italy). The absence of natural metastrongyloid infections was confirmed by artificially digesting and examining a representative number (i.e., 10%) of snails. During the maintenance, gastropods were placed in plastic boxes filled with natural soil, covered with a net and kept at 23±1°C. Snails were fed every two days with lettuce *ad libitum*. First-stage larvae of *A*. *abstrusus* and *T*. *brevior* used for infection were obtained from cats artificially infected with pure isolates, originating from Hungary and Italy, respectively. Two groups of 50 snails each were prepared, being infected with ~500 L1 of *A*. *abstrusus* and *T*. *brevior*, respectively, as described in [21]. Snails were then placed into two different boxes, according to lungworm species, and two specimens from each group were artificially digested at +6, +12 and +18 days post infection (dpi) to evaluate the success of the infection.

### Elimination of third-stage larvae via mucus

In order to assess whether larvae are released in the mucus of snails, six groups (G1–G6) of 4 snails each were formed 20 dpi for each metastrongyloid species. Snails were placed into a 1 l plastic box for bacteriological use, together with 25 ml of tap water, and subjected to different stimuli. Specimens in G1 and G4 were left with water, those in G2 and G5, and G3 and G6 were fed with lettuce (3 g) or commercial cat food (0.8 g), respectively. Group 1, G2 and G3 were placed on a thermostat shaker, applying approximately 50 oscillations per minute overnight, while G4, G5 and G6 were constantly kept without mechanical stimuli. All snails were housed under controlled room temperature (23±1°C). Once a day in the morning for 20 days, snails were taken out from their plastic boxes, which were rinsed with 20 ml of tap water (30°C), and the solution was filtered through a 180-μm meshed sieve. The filtrate was centrifuged at 600 *g* for 5 min and the pellet examined under a light microscope. Finally, snails were returned to the same experimental group boxes. Larvae recovered were quantified and identified using appropriate morphological keys [15,21].

### Release of third-stage larvae in water

In order to evaluate the emergence of larvae from dead submerged snails, at the end of the experiment described above, gastropods were identified with a letter, placed into a 50 ml tube, submerged in tap water and left at room temperature (23±1°C). Every 24 h for three consecutive days, the sediment was collected and centrifuged at 600 *g* for 5 min, and the pellet was examined under a light microscope. Nematode larvae were considered motile if they were not damaged and were moving within 10 s [22].

### Artificial digestion and larval molecular identification

Each snail specimen was artificially digested in a solution of 1% HCl (100 ml) and 1.2 g powdered pepsin (Sigma-Aldrich, St. Louis, Missouri, United States), as described elsewhere [21]. The solution was collected in plastic tubes and centrifuged at 600 *g* for 5 min. The whole suspension (5 ml) was examined under a light microscope; larvae were morphologically identified according to species and developmental stage and counted.

Ten representative larval specimens of each of *A*. *abstrusus* and *T*. *brevior* were isolated from the sediment of digested snail tissues and stored in plastic vials, containing phosphate buffer saline (PBS) at −20°C to be analysed by PCR. DNA was extracted using the DNeasy Blood & Tissue Kit (Qiagen, GmbH, Hilden, Germany), in accordance with the manufacturer instructions, and a duplex-PCR was performed, using species-specific forward primer sets, amplifying ITS-2 region of different size (i.e., *A*. *abstrusus*: 220 bp; *T*. *brevior*: 370 bp) [11]. Sequences were determined from both strands and compared with those available in the GenBank database by Basic Local Alignment Search Tool (BLAST).

### Statistical analysis

The arithmetic mean of L3 counted in the mucus, in water and in sediment following artificial digestion was calculated for each group. Larval counts obtained from these three experiments were totalled for each snail and the infection rate was calculated using the following formula: infection percentage = (500—T)/500, where T was the total number of L3 recovered. Accordingly, the values were compared between *A*. *abstrusus* and *T*. *brevior* infected snails using the Mann-Whitney U-Test. The statistical analysis was two-sided, at the significance level *p* = 0.05.

### Histological examination of snails

To assess the anatomical localization of metastrongyloid larvae, two infected snails were examined every three days, from +1 to +30 dpi (i.e., 11 time points). Snails were anesthetized with menthol steam in a plastic box for 3–5 hours and, as soon as the foot was completely extended and insensitive to touch, they were deprived of their shells and fixed in a 50 ml vial with 10% neutral buffered formalin solution, to be histologically examined. Longitudinal sections across the middle of the body and parasagittal sections through the coiled part of the snail were routinely processed, embedded in paraffin and 5 μm slices were stained with haematoxylin and eosin (H&E). The presence of a tissue inflammatory response around larvae was also recorded.

## Results

No larval nematodes were recovered from *H*. *aspersa* specimens digested before the infection. Conversely, larval nematodes were recovered from all snails experimentally infected with either *A*. *abstrusus* or *T*. *brevior* (n = 6 for each parasite) at each sampling point.

Third-stage larvae of both feline lungworm species were detected in the mucus of snails in all groups, except G1; only a single L2 of *T*. *brevior* was recovered from G4 at 20 dpi (Table 1). In particular, out of 37 *A*. *abstrusus* (mean 6.2±12.4) and 19 *T*. *brevior* (mean 3.2±7.3) L3 recovered, the highest number of larvae was observed in G2 and G5 for both lungworm species. Overall, 7.8% and 1.8% of *A*. *abstrusus* and *T*. *brevior* L3, respectively, were recovered in the mucus without significant difference recorded in the total number of L3 between *A*. *abstrusus* and *T*. *brevior* groups (*p*>0.05).

**Table 1:** Group and total number of *Aelurostrongylus abstrusus* and *Troglostrongylus brevior* larvae detected in the mucus of *Helix aspersa* snails or in the water solution where gastropods had died.

	Larvae in the mucus		Larvae in water	
Groups (Stimulus)	*A*. *abstrusus*	*T*. *brevior*	*A*. *abstrusus*	*T*. *brevior*
G1 (Water and stirring)	-	-	18	4
G2 (Lettuce and stirring)	9	12	12	4
G3 (Cat food and stirring)	2	1	27	14
G4 (Water)	7	1*	18	6
G5 (Lettuce)	13	4	34	0
G6 (Cat food)	6	2	63	11
Total	37	20	172	39

*Second-stage larva

In snails that had died through submersion, larvae of *A*. *abstrusus* (n = 172; mean 7.2±12.2) and *T*. *brevior* (n = 39; mean 1.6±2.6) were detected in the sediment of 21/24 (87.5%) and 10/24 (41.7%) specimens, respectively (Tables 1 and 2); the mean number of *A*. *abstrusus* L3 was significantly higher (*p* = 0.00076). In total, 5.7% *A*. *abstrusus* and 2.7% *T*. *brevior* L3 were found in the water (Table 1).

**Table 2:** Total number and percentage of *Aelurostrongylus abstrusus* and *Troglostrongylus brevior* L3 detected in each snail specimens from different groups following water flooding and digestion examinations.

		*A*. *abstrusus*			*T*. *brevior*		
Groups (Stimulus)	Specimen	Flooding	Digestion	Total L3	Flooding	Digestion	Total L3
G1 (Water andstirring)	A	-	271	271	4	17	21
	B	6	72	78	-	30	30
	C	8	98	106	-	170	170
	D	4	78	82	-	47	47
G2 (Lettuce andstirring)	A	1	66	67	-	98	98
	B	7	216	223	-	56	56
	C	3	184	187	-	158	158
	D	1	14	14	4	50	54
G3 (Cat foodand stirring)	A	7	156	163	8	68	76
	B	12	91	103	-	39	39
	C	3	23	26	6	105	111
	D	5	98	103	-	43	43
G4 (Water)	A	5	386	391	-	50	50
	B	10	98	108	3	71	74
	C	2	73	75	3	55	58
	D	1	19	20	-	41	41
G5 (Lettuce)	A	1	67	68	-	55	56
	B	20	95	115	-	64	64
	C	5	77	82	-	10	10
	D	8	145	151	-	30	30
G6 (Cat food)	A	2	323	325	1	19	20
	B	60	49	109	2	39	41
	C	-	86	86	-	57	57
	D	1	73	74	8	18	26
**Total**		**172 (5.7%)**	**2858 (94.3%)**	**3030**	**39 (2.7%)**	**1390 (97.3%)**	**1429**

**Fig 1:**
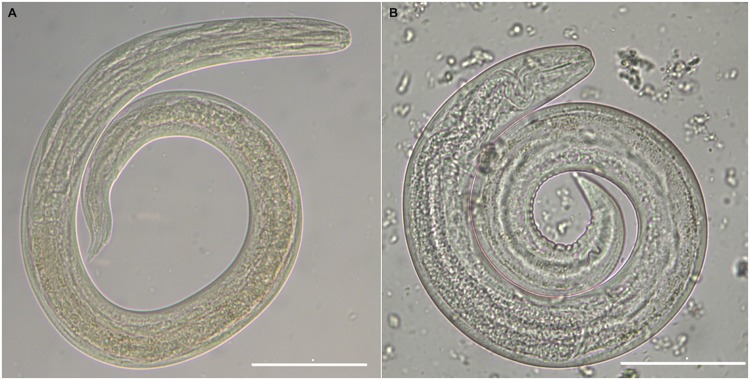
Infective L3 of *T*. *brevior*. (A) and *A*. *abstrusus* (B) detected in the mucus of *H*. *aspersa* snails at 25 days post-infection (scale bar = 50μm).

Following the artificial digestion, all *H*. *aspersa* snails scored positive for metastrongyloid larvae. A total of 2858 *A*. *abstrusus* and 1390 *T*. *brevior* L3 were recovered and individual larval counts are reported in Table 2. The mean number of L3 was 127.8 (min 14–max 386) for *A*. *abstrusus* and 60.3 (min 10–max 170 L3) for *T*. *brevior*. Taking into account the original 500 L1 administered to each snail and the total number of larvae recovered, the proportion of L1 that had moulted to L3 was 25.6% for *A*. *abstrusus* and 12.1% for *T*. *brevior* (see statistical analysis paragraph). All nematodes detected were actively motile, and all specimens were morphologically identified as infective L3, since they had lost their outer sheaths and measured 442.7±17.8 μm (i.e., *T*. *brevior* and Fig 1A) and 548.6±30.3 μm (i.e., *A*. *abstrusus* and Fig 1B). This identification was confirmed by molecular amplification and sequencing of partial ITS-2 gene. Nucleotide sequences, examined by BLAST, displayed a 100% homology with those of *A*. *abstrusus* and *T*. *brevior* in GenBank (accession numbers KF751656 and KF751655).

**Fig 2:**
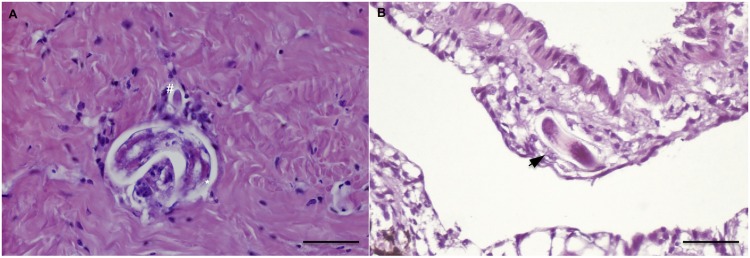
Histopathology: Free larvae of *A*. *abstrusus* (21 dpi) in snail foot observed in transverse (hash) and oblique sections (star) in the fibro-muscular tissues. (A); oblique larval section of *T*. *brevior* (9 dpi) the subpallial tissue (arrow) (B) (scale bar = 50μm; H&E).

During the histological examination, larvae of metastrongyloids were found at different time points, starting from 1 dpi and 9 dpi for *A*. *abstrusus* and *T*. *brevior*, respectively. Larval transverse sections were recognized as belonging to *A*. *abstrusus* or *T*. *brevior* according to their mean diameter, detected on round bodies, measuring ~25 and 20 μm, respectively. The number of snail sections positive for *A*. *abstrusus* (n = 103) was higher than that for *T*. *brevior* (n = 16), with a mean number of 6.4 for the former and 1.0 for the latter species. For both metastrongyloids, larvae were mainly observed in the anterior and posterior parts of fibro-muscular tissue (Fig 2A) of the foot and in the skirt, close to the pedal and oesophageal glands. Larvae were randomly detected in other organs, such as the kidney parenchyma, the wall of the pallial cavity and the connective sub-epithelial layer of the intestine (Fig 2B). Nonetheless, free larvae were rarely found in infected snails at different time points (i.e., at 1, 12 and 21 dpi for *A*. *abstrusus* and at 9 dpi for *T*. *brevior*). Specimens were localized in the fibro-muscular tissue of the foot, with some of them near vessels, and one specimen in the coelom. All individuals were separated from the surrounding tissue by a thin optically empty space.

**Fig 3:**
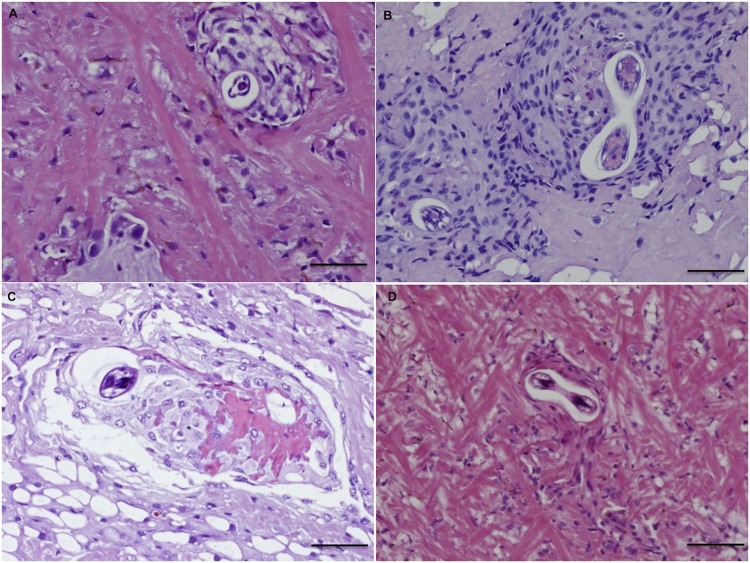
Histopathology: Inflammatory response to *A*. *abstrusus* in the *H*. *aspersa*. Cell-poor granuloma formation with vacuolated amebocytes at 3dpi. (A); cell-rich granuloma formation at 9 dpi (B); small necrotic granuloma at 15 dpi (C); fibroblast-like reaction at 27 dpi (D) (scale bar = 50μm; H&E).

**Fig 4:**
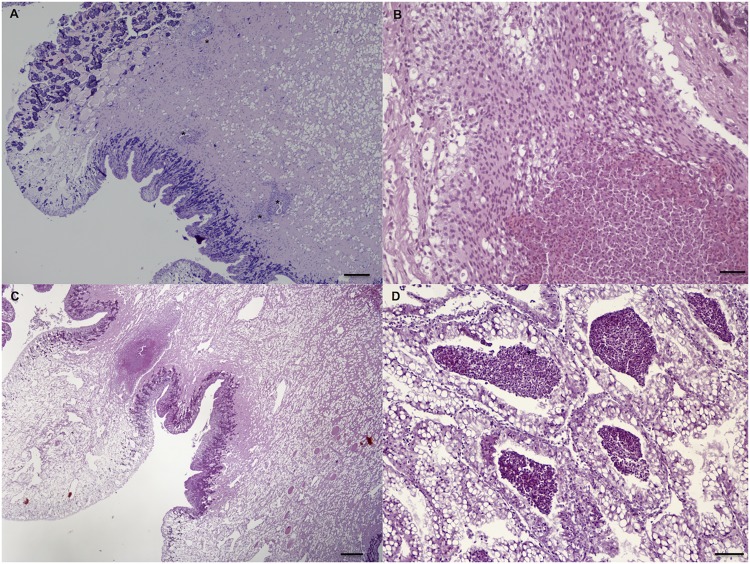
Histopathology: Larvae of *A*. *abstrusus* (asterisks) in the snail foot at 9 dpi, within granuloma formations. (A); large necrotic granuloma with peripheral well-preserved amebocytes and their debris in the centre in snail infected by *T*. *brevior* at 21 dpi (B); polipoid enlargement of the ventral surface of the foot with dilated vessels and an abscess-like formation in *T*. *brevior*-infected snail (21 dpi) (C); multifocally-distributed amebocytes aggregates in the kidney parenchyma in *T*. *brevior*-infected snail (3 dpi) (D) (scale bar = 50μm; H&E).

The tissue response to nematode larvae included: *i*) cell-poor (3 dpi; Fig 3A) and cell-rich (9 dpi; Fig 3B) granuloma-like formations, composed of non-vacuolated or vacuolated epithelioid amebocytes; *ii*) small necrotic granulomas (15 dpi; Fig 3C); *iii*) fibroblast-like encapsulations (27 dpi; Fig 3D). The severity of the inflammatory pattern ranged from mild reactions, featured by vessel dilatation, mild increase of the cellularity and small granulomas (Fig 4A), to strong focal reactivity. In the latter case, large necrotic granulomas were characterized by nodular aggregates of amebocytes in the periphery and their debrided remnants in the centre (Fig 4B). This last response was mainly observed in *T*. *brevior* infected snails, which were often featured by enlarged ventral surfaces of the foot and prominent vessel dilatation, along with few larval granulomas (Fig 4C). The occurrence of large amount of amebocytes was also seen in the kidney of *T*. *brevior* snails at 3 dpi (Fig 4D).

## Discussion

Results of this study indicate that L3 of *A*. *abstrusus* and *T*. *brevior* are either shed in the mucus by live terrestrial gastropods or released into the environment when snails had died in water. Both of these pathways of L3 elimination may represent alternative route(s) of transmission for feline lungworms under field conditions.

The emergence of *A*. *abstrusus* and *T*. *brevior* larvae from living snails, without either mechanical stressors or lack of food, indicates that this can occur spontaneously. This phenomenon has also been shown for other metastrongyloids [23]. Indeed, *A*. *costaricensis* L3 may leave *Biomphalaria glabrata* freshwater snails, as well as *Phyllocaulis soleiformis* and *Sarasinula marginata* slugs [24–26]. Similarly, *A*. *cantonensis* has been detected in the mucus of *Parmarion martensi* semi-slugs [7] and larvae of the muskoxen lungworm *Umingmakstrongylus pallikuukensis* (Strongylida, Protostrongylidae) emerged from *Deroceras* slugs and *Catinella* snails, being both found in the sediment of uneaten gastropod food or in water, coated with snail mucus [27]. In addition, *Angiostrongylus vasorum* (Strongylida, Angiostrongylidae) L3 may abandon *B*. *glabrata* gastropods if exposed at different temperatures [28]. Interestingly, when infected snails are given food, the elimination of L3 increases, probably as the result of enhanced mucus secretion [19]. Accordingly, the contamination of cat food left outdoors by snails shedding L3 might be a significant route of infection. This is, however, less likely to be the case when cats ingest grass or vegetables (e.g., the catnip *Nepeta cataria*) to induce vomiting as a remedy of hairballs.

Again, the elimination of *A*. *abstrusus* and *T*. *brevior* larvae by dead snails into water represents a further route of transmission for both lungworms, as corroborated by previous findings on *A*. *cantonensis* and *A*. *costaricensis* outbreaks, which most likely had occurred following the consumption of contaminated vegetables [20,29,30]. Indeed, considering that cats may also drink mouldy water, the swallowing of L3 cannot be ruled out. Although the longevity of L3 that have emerged from snails has not been assessed for both feline lungworms, it has been estimated to be up to 72 h for *A*. *cantonensis* from land snail [30] and seven days from water snails [24]. Although *in vivo* models documenting the above mentioned pathways are not available for feline lungworms, the transmission of free-living metastrongyloid larvae from freshwater snails to the final host has been demonstrated for *A*. *cantonensis* [7,29] and *A*. *vasorum* [28].

Whether the shedding/elimination of L3 represents an active or a passive process (i.e., triggered by the metastrongyloid itself or primed by gastropod hosts) is yet to be determined. However, considering that all *A*. *abstrusus* and *T*. *brevior* larvae seen here were L3 without any larval sheath, they are likely to have actively left their hosts, once reached the infective stage. Indeed, L3 of metastrongyloids are characterised by being motile, which enables them to move through the host (i.e., from the gut to the small intestine and blood stream) [12]. This hypothesis is supported by data on *U*. *pallikuukensis*, whose larvae do not emerge from the intermediate gastropod hosts, until they have reached the infective stage around three weeks post infection [25].

Results of the histology confirm that lungworm larvae can be found either in the foot and the viscera of infected specimens [31]. The localization of L3, mainly in the fibro-muscular layer of the foot, was probably due to the massive vascular supply in this organ, which provides the best conditions for the homing of the parasites, as already suggested for *A*. *costaricensis* [32]. Based on the data herein recorded, the intrinsic mechanical action of foot fibro-muscular layer might also account for the release of the L3 into the external habitat, along with the mucus secretions. Whether the detection of L3 in the foot represents a primary site of entrance for L1 throughout the skin (see [32,33]), or it occurs following the ingestion and vascular dissemination from other organs (e.g., kidney or skin), could not be assessed.

Even if the histology cannot be considered the best method to quantify the number of larvae in snails, L3 of *A*. *abstrusus* were more frequently detected in all experiments herein carried out than those of *T*. *brevior*. Therefore, the small number of *T*. *brevior* larvae might be accounted for by the presence of numerous large necrotic granulomas in snails, which most likely represented a first defence of the gastropod against the larval invasion. This pattern may indicate that *T*. *brevior* is more harmful for *H*. *aspersa*, and therefore less adapted to this intermediate host than *A*. *abstrusus*. This may be also inferred by the higher moulting percentage of *A*. *abstrusus* L3 (i.e., 25.6%) than *T*. *brevior* (i.e., 12.1%) under the same laboratory conditions. This hypothesis requires to be verified for other snail species, but it may explain the wider geographical distribution and higher prevalence of *A*. *abstrusus* infection in feline populations [9,34]. Further studies are required to assess the interactions between the nematode and the gastropod, in order to provide a more complete picture of the influence of host species on the epidemiology of these lungworms, as already carried out for schistosomosis [2]. Although several studies have reported the host range for metastrongyloid hosts [15], few have assessed the relative susceptibility of gastropods for those pathogens.

Nonetheless, based on the findings documented here, the existence of competitive exclusion between the two lungworms in the same intermediate host cannot be ruled out. Indeed, the competitive exclusions principle, also known as Gause’s law, says that if one of two species has even the slightest advantage over the other, that species will dominate [35]. Gause’s law has already been demonstrated for several parasite—host systems (e.g., human filarioids, livestock haemoparasites, *Taenia* cestodes) [36]. Accordingly, it has been demonstrated that *Schistosoma mansoni* and *Schistosoma rodhaini* trematodes display a species-specific mate preference in *Mus musculus* mice, with males of the latter species being dominant over those of the former [37]. Importantly, the inter-specific competition has been implicated with an increase in parasite virulence [37]. However, since none of these models has ever been applied to feline metastrongyloids, studies on snail immunology could provide useful data to help demonstrate whether gastropods are more susceptible to particular metastrongyloid species.

### Concluding remarks

Further large-scale epidemiological studies that will help identify risk factors associated with the spread of *A*. *abstrusus* and *T*. *brevior* are needed. Based on data herein reported, owners should pay attention to their cats living outdoor and clean the water and food bowls if left outside, considering that snails and slugs may shed L3. In addition, since *H*. *aspersa* snails may act as intermediate hosts for other metastrongyloid nematodes, such as *Oslerus rostratus* (Strongylida, Filaridae) [38] or *A*. *vasorum* [12], the potential shedding of both lungworms within gastropod mucus should be investigated. Therefore, the identification of alternative pathways for parasite transmission is important particularly since the geographic range of gastropod-borne diseases is expanding due to *inter alia* introduction of allochthonous infected snails [39,40].
